# Targeting MYCN in Molecularly Defined Malignant Brain Tumors

**DOI:** 10.3389/fonc.2020.626751

**Published:** 2021-01-28

**Authors:** Anna Borgenvik, Matko Čančer, Sonja Hutter, Fredrik J. Swartling

**Affiliations:** ^1^ Department of Immunology, Genetics and Pathology, Science for Life Laboratory, Rudbeck Laboratory, Uppsala University, Uppsala, Sweden; ^2^ Department of Oncology-Pathology, Karolinska Institutet, Stockholm, Sweden

**Keywords:** MYCN, brain tumor, targeted therapies, c-MYC, OCT4, medulloblastoma, glioma

## Abstract

Misregulation of MYC genes, causing MYC overexpression or protein stabilization, is frequently found in malignant brain tumors highlighting their important roles as oncogenes. Brain tumors in children are the most lethal of all pediatric malignancies and the most common malignant primary adult brain tumor, glioblastoma, is still practically incurable. MYCN is one of three MYC family members and is crucial for normal brain development. It is associated with poor prognosis in many malignant pediatric brain tumor types and is focally amplified in specific adult brain tumors. Targeting MYCN has proved to be challenging due to its undruggable nature as a transcription factor and for its importance in regulating developmental programs also in healthy cells. In this review, we will discuss efforts made to circumvent the difficulty of targeting MYCN specifically by using direct or indirect measures to treat MYCN-driven brain tumors. We will further consider the mechanism of action of these measures and suggest which molecularly defined brain tumor patients that might benefit from MYCN-directed precision therapies.

## Introduction

The development of massive sequencing efforts and molecular profiling of malignant brain cancer biopsies from patients and the strive to characterize them better has transformed the diagnosis of these tumors (1–5). The augmented conception that malignant brain tumors could no longer be defined as a rather small selection of histologically defined entities but in fact comprise over a hundred different molecular subgroups, suggest it is time for a change in how treatment could be more specialized and tailored. The generation of more clinically relevant models recapitulating such subgroups, including MYCN-driven brain cancers have helped improved our understanding how these biologically distinct tumors can be efficiently targeted. Recent single-cell sequencing technologies can help to further recognize the heterogeneity of the brain cancer (6, 7). Altogether, this can improve therapies, risk-stratification schemes and reduce recurrences, which are usually fatal for these types of tumors.

Here, we will describe the prevalence of MYCN alterations in malignant brain cancer in children and adults. We will also portray current treatment regimens and patient outcomes and reflect on how targeted treatments of MYCN would improve future therapies for the most common and aggressive types of brain tumors. In order to develop such targeted strategies, we must first define what we have learned from the biological properties and regulation of MYCN in normal and malignant cells. We will specifically address what molecular information we can use from appropriate cell systems and animal models of brain cancer in order to develop better MYCN-targeted treatments.

Brain cancer as compared to other tumors outside of the central nervous system (CNS) present an obvious hallmark. They reside in a partially protected compartment that implicates difficulties and complications concerning drug delivery and penetrance over the blood-brain barrier (BBB). The difference in childhood and adult tumors is still evident as well as the fact that treatment can affect normal brain development and can cause severe long-term side effects. The translational transfer of basic molecular findings from the bench into reliable, tailored drugs for these patients to the bed-side is thus not always straight-forward and requires careful selection and testing.

## Diagnosis and Molecular Profiling of Brain Tumors With MYC Family Activation

Brain and other CNS tumors are the most common solid tumors in children and the most common cause of pediatric cancer death. Gliomas are the most common brain tumors in children, with the majority being low-grade gliomas (LGGs). The most frequently diagnosed single histological type of tumor is pilocytic astrocytoma, which accounts for 18% of primary brain tumors in children ages 0–14 years (8). These tumors are clinically classified as WHO grade I and are almost always associated with single genetic alterations in the RAS/MAPK pathway (9, 10).

**Figure 1:**
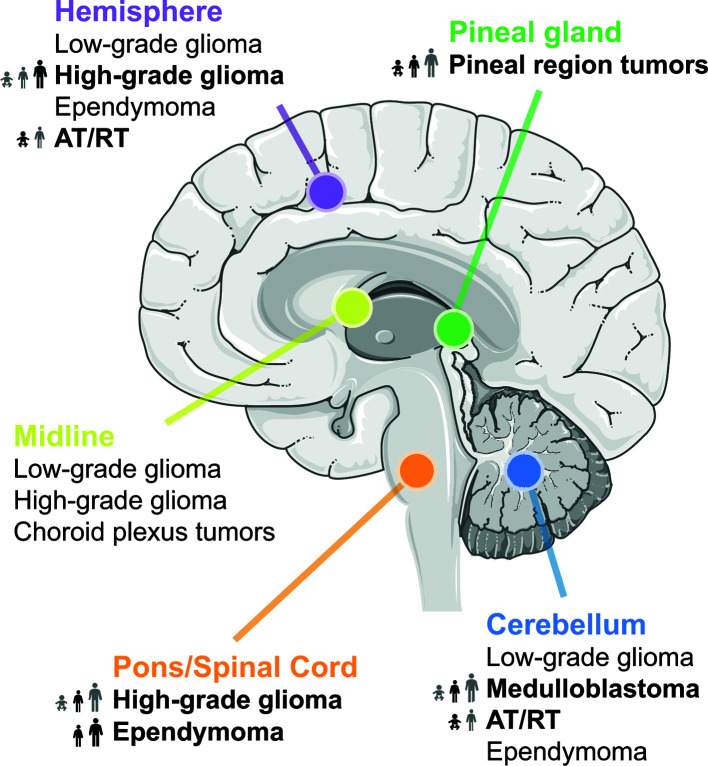
Location of common brain tumors. Tumor entities with known MYC involvement are highlighted in bold and their frequency across all age groups is indicated.

Meningiomas, pituitary tumors, and malignant gliomas are among the most common primary adult brain tumors (11) Primary brain tumor incidence is seven to eight times higher in adults as compared to children in the United States (8). Here, non-malignant brain tumors are overall more than twice as common as malignant brain tumors. This review will focus on the most common types of malignant primary brain tumors in children and on primarily malignant gliomas in adults (**Figure 1**).

### High-Grade Gliomas in Children

Pediatric high-grade gliomas (pHGGs) account for approximately 17% of all pediatric CNS tumors (8). pHGGs are a histologically heterogeneous group of tumors with the most frequent types being anaplastic astrocytoma (WHO grade III) and glioblastoma (GBM) (WHO grade IV). The outcome for pHGGs as a whole is poor with 5-year survival rate of 20% (12). In general, HGGs in children are biologically distinct from their adult counterparts. Molecular profiling of large cohorts of pHGG patients resulted in discovery of several genetic and epigenetic subtypes (13). Important molecular features of pHGGs include recurrent mutations in genes encoding the histone variants H3.3 and H3.1 with the mutations K27M or G34R/V defining distinct epigenetic subgroups. The last update (2016) of the WHO classification of CNS tumors recognizes established molecular variants of HGG including IDH-wildtype and -mutant GBM, as well as H3.3/H3.1 K27-mutant diffuse midline glioma, which were formerly known as diffuse intrinsic pontine glioma (DIPG). The latter group is associated with the most dismal outcome with less than 10% of patients surviving beyond 2 years (14). The H3.3 G34 subtype pHGGs typically occur in the cerebral hemisphere and upregulated MYCN expression has been observed in this subgroup (15). Mutations in IDH1, which are frequent in adult gliomas, are only found in a small proportion of pHGGs (16). Among the remaining approximately 50% of tumors that lack histone H3 and IDH1 mutations (H3/IDH1-WT) several subgroups are emerging. One biologically very aggressive subtype is characterized by enrichment of MYCN amplifications, whereas other subgroups are enriched for amplification in receptor tyrosine kinase genes PDGFRA or EGFR (17). Finally, a specific malignant type of spinal ependymoma in older children and adults with poor prognosis and a propensitiy to metastasize, has recently been shown to contain MYCN amplifications (18).

### High-Grade Gliomas in Adults

Due to its critical role in regulating cell cycle and metabolism, MYC has been found overexpressed in GBM, with a tendency towards correlation of astrocytic GBM grade with the level of both nuclear and cytoplasmic MYC (19–21). In addition to increased immunostaining, authors also demonstrated positive correlation of astrocytoma grade with the number of MYC copies. MYCN overexpression and amplification have also been frequently associated with GBM (in about 40% of tumor samples) (22, 23).

IDH1 mutation is a known predictor of response to temozolomide (24) and conveys sensitivity to metabolites of alkylating agents. In a subset of IDH1 mutant GBM, Odia et al. found a correlation with MYC expression (25), indicating MYC status as an adverse prognostic factor for IDH-mutant GBM.

In malignant glioma with primitive neuroectodermal components (MG-PNET), a rare type of brain tumor that most likely develops from already existing glioma, about half of the patients demonstrate mutually exclusive MYC or MYCN amplifications (26).

MYCN was early found to form extrachromosomal double minutes in neuroblastoma (NB) (27). Recent sequencing efforts show that both MYC and MYCN frequently form extrachromosomal amplifications in GBM (28, 29). Accumulation of such extrachromosomal DNA is essentially connected to tumor evolution and is associated with overall poor prognosis in cancer (30).

### Embryonal Tumors in Children

CNS embryonal tumors, including medulloblastoma (MB) and atypical teratoid/rhabdoid tumors (ATRT), account for 13.1% of primary CNS tumors in children (8). Nearly two-thirds of embryonal tumors are diagnosed as MB, which is the most frequent malignant brain tumor of childhood. Integrative genomic studies have shown that MB is not a single entity, but rather a heterogeneous group with distinct clinical and biologic features (6). Molecular subgrouping of MB into WNT, SHH, Group 3 and Group 4 tumors, was integrated in the most recent WHO classification and is currently used for risk stratification replacing diagnosis and treatment of these entities by histopathology. MYC amplifications are the most frequently observed driver events in Group 3, whereas MYCN is overexpressed or amplified in SHH subtype and some Group 4 MBs (3, 31). ATRTs are a variant of embryonal brain tumors occurring predominantly in very young children. Despite sharing the common genetic hallmark of mutations in SMARCB1, recent studies have revealed three distinct subgroups (TYR, SHH, MYC) based on methylation and gene expression data (32). MYC overexpression is the marker of the ATRT-MYC subtype, which is comprised of mostly supratentorial tumors.

### Pineal Brain Tumors in Children and Adults

Additionally, a MYC-subgroup has recently been identified in pineoblastoma, a rare but quite frequently metastatic, pediatric brain tumor of the pineal gland with modest overall survival despite intensive therapy (33). Interestingly, while pineoblastoma usually present with molecular profiles distinct from medulloblastoma some embryonal tumors identified as pineoblastoma in the pineal region were recently identified as WNT-driven medulloblastomas using methylation profiling (34).

## Current Treatment of Brain Tumors

There is no current international consensus on the treatment of neither pediatric nor adult brain tumors. However, most patients see surgical tumor resection, radiotherapy, and chemotherapy. Factors such as diagnosis, grade, location, tumor dissemination, and age impact how each of these parts are implemented in the treatment plan. Surgical removal of the tumor mass is always included when it is possible to do so. Successful surgery depends on how much of the tumor can be safely resected and is the biggest prognostic factor for overall survival and deciding subsequent radiotherapy and chemotherapeutic regimens.

Radiation therapy is often given as a high dose fractioned over several occasions and directed at the primary tumor site. Patients with spinal metastases receive radiation therapy to the entire cranio-spinal axis. For adult brain tumors, radiotherapy is a major part of standard treatment. In children, MB patients have great benefits on survival from irradiation (35) and evidence points to that conclusion also for ATRT tumors (36). Despite the detrimental side effects radiation therapy has on young patients it is rarely omitted from treatment unless the patient is younger than 3–4 years. MB can be further stratified to identify high-risk MYC/MYCN overexpressed tumors (37). Those patients that would commonly receive a higher dose of radiotherapy (38).

The youngest patients with high-grade brain tumors that are not eligible for radiation and/or surgery are especially negatively affected by the lack of efficient and safe chemotherapeutics. Tumor treating fields is a low toxicity, non-invasive, non-pharmacological treatment of both newly diagnosed and recurrent GBM, used in combination with standard therapy. It is electromagnetic fields administered through the skin of the scalp and its arrangement is individualized to optimize effect at the tumor site. As it is suggested to target primarily dividing cells during mitosis and causes DNA damage in cycling cells, normal cells in the brain should be spared (39). Data also suggest that tumor treating fields is both safe and feasible in pediatric patients (40).

Many chemotherapeutics have been used empirically for decades despite showing substantial effects on prolonging survival of brain tumor patients. On the other hand, targeted therapies for primary brain tumors have of yet not lived up to the expectations and some of these lead to treatment resistance in recurrent tumors. Due to the inability of current treatment options to cure or even extensively prolong survival of patients, both adult and pediatric patients are often enrolled in multinational clinical trials. Careful stratification of patients into correct molecular subgroups and repeated biopsying (41) could help improving the success rate of targeted therapies.

Immunotherapies in brain cancer is a rapidly emerging field. Checkpoint inhibitors have been intensively tested but unfortunately shown limited efficacy in glioblastoma patients (42). It is evident that immunological responses need to be increased in these patients in order to show better effects. MYC is known to suppress checkpoint proteins PD-1 and CD47 (43) and MYC inhibition is found to re-express these proteins making these immunotherapies effective again (44). Recent animal studies further suggest that p53 depletion is suppressing major histocompatibility complex (MHC) class 1 presentation, which mediates T cell immune escape in MYC-driven medulloblastoma (45).

Chimeric antigen receptor (CAR) T cell transfer is an interesting option in pediatric brain tumor patients (46) as long as severe side effects of cytokine release can be carefully managed and avoided. It is further known that delivery of CAR T cell therapies into the cerebrospinal fluid compartment could provide a better chance for this treatment to reach the tumors. Such an approach has shown promising results when tested in animals using PDX models of medulloblastoma (47) and many clinical trials for children with brain tumors are currently ongoing and under evaluation.

Finally, dendritic cells are important antigen-presenting cells that express both MHC class 1 and 2 molecules and can stimulate antitumor immune responses. Dendritic cell vaccines (48) are currently tested in clinical trials for GBMs and has shown rather promising results as they may increase survival for these patients (49).

## MYCN Biology and Regulation in Normal Cells

**Figure 2:**
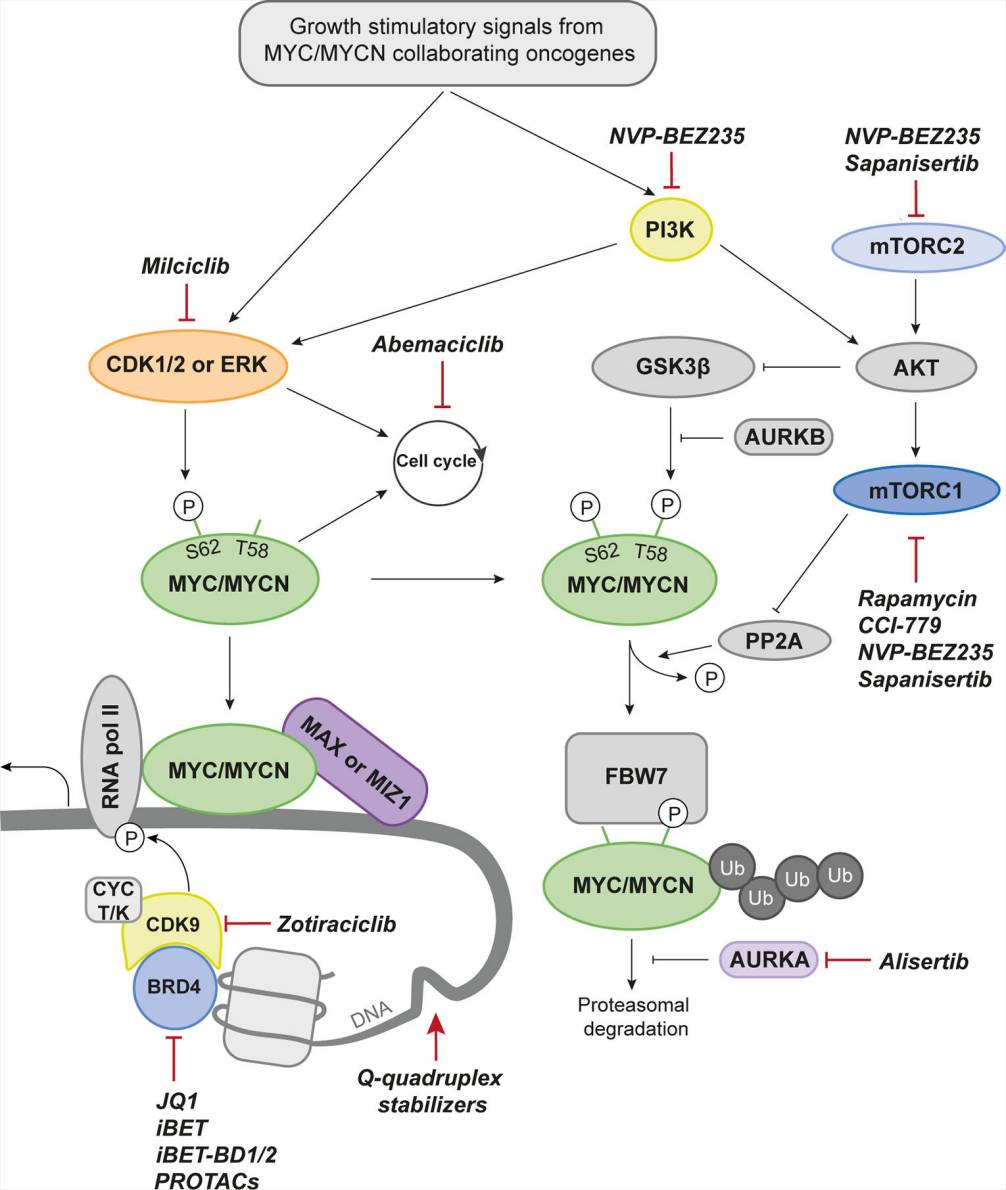
Drug Targets in MYCN Biology. Proteins that can be targeted pharmacologically and discussed in the current review are marked in color. Drugs with preclinical data are denominated with group affiliation. A selected set of drugs that have reached the clinical stage (either approved or in clinical trials for the indicated target) are named. CDK1/2, Cyclin dependent kinase 1/2; MAX, MYC-associated factor X; MIZ1, MYC-interacting zinc-finger protein 1; RNA pol II, RNA polymerase II; BRD4, Bromodomain-containing protein 4; CYC T/K, Cyclin T/K; PROTACs, Proteolysis targeting chimeras; PI3K, Phosphoinositide 3-kinase; AURKB, Aurora kinase B; FBW7, F-box and WD repeat domain-containing 7; Ub, Ubiquitin; AURKA, Aurora kinase A; mTORC2, Mammalian target of rapamycin complex 2; mTORC1, Mammalian target of rapamycin complex 1; AKT, Protein kinase B; PP2A, Protein phosphatase 2; P, phosphorylation.

The family of MYC proteins (c-MYC, MYCN, and MYCL) are basic helix-loop-helix-zipper (bHLHZ) transcription factors, tightly regulated by extracellular growth stimulatory signals and an intricate intrinsic mechanism behind expression, activation, and degradation of MYC proteins (**Figure 2**). The MYC family of transcription factors binds Enhancer BOX (E-BOX) sequences to promote or repress transcription of its targets. This is enabled and coordinated when the MYC protein heterodimerizes with MYC Associated Factor X (MAX) and together bind to the E-BOX (50). Around 20,000 E-BOX sequences are found in the human genome why MYC is often referred to as a transcriptional master regulator.

MYC and MYCN siblings are similar in structure, and can often substitute each other’s functions (51). MYC proteins are often redundant in cancer and showing mutually exclusive expression patterns of MYC and MYCN in patient samples (52, 53). Repression of target genes by MYC proteins involves another co-factor, MYC interacting zinc finger 1 (MIZ1) that tethers MYC-MAX into a ternary complex to promoter regions of negative cell cycle regulators like CDKN1A or CDKN2B (54, 55). Still, there are important differences in how MYC members interact with certain co-factors including MIZ1 (56) and regulate signaling pathways, revealing an increased complexity in how to target these factors using direct or indirect therapies. It is also recognized that MYC proteins interact with chromatin modifying co-factors in order to remodel chromatin structure close to their binding sites (57).

The transcriptional output signature of MYC is highly dependent on the cellular context. The different MYC family members are very similar but MYCL and MYCN are distinctly expressed in specific tissues (lung and neuronal tissue, respectively) unlike c-MYC which is found expressed in most tissues. MYCN is crucial for normal brain development (58, 59).

A proliferating cell would allow stabilization of the MYC proteins while a quiescent cell quickly degrades the proteins through the ubiquitin degradation pathway, dependent on the E3 ubiquitin ligase FBW7. Two phosphorylation sites play major roles in the life cycle of MYC. These are serine 62 (S62) and threonine 58 (T58) (60). Consecutive phosphorylation and dephosphorylation at these sites govern the activity, stability, and degradation of the protein (61). Several proteins related to mitogenic signaling are associated with or directly phosphorylates MYCN at serine 62 to stabilize the protein. Among those are mitogen-activated protein kinases (ERKs) and cyclin-dependent kinases (CDKs), both important for cell growth and proliferation (60, 61). Phosphorylation of this residue causes a conformational change from *cis* to *trans* which increases the affinity for DNA binding and subsequent transcriptional activity of MYC. On the other hand, it also makes it recognizable by glycogen synthase kinase 3 beta (GSK3β), a kinase that will phosphorylate MYC at T58 (60). Mutations at this site will lead to MYC protein stability and can also cause MYCN-driven MB emphasizing the importance of this key event in MYC regulation (62). This is the start of the MYC degradation process and is followed by dephosphorylation of S62 by protein phosphatase 2 (PP2A) that only binds MYC when both sites are phosphorylated (63). The E3 ubiquitin ligase F-box and WD repeat domain-containing 7 (FBW7) recognizes phosphorylated T58 and sentence MYC to proteasomal degradation (64). In normal cells this is a well-functioning machinery tightly controlled at all levels to avoid neoplastic development. The last resort of safety checks is MYC’s ability to promote apoptosis when expressed at high levels (65, 66). In cancer, however, it is often overcome by mutations in proapoptotic pathways including p53. This will allow uncontrolled effects of MYC overexpression and activation leading to rapid proliferation and tumor formation.

## Direct or Indirect Targeting of MYCN in Brain Tumors

### Genetically Engineered Proof-of-Concept Inhibition Models of MYCN

MYC proteins play an important role in oncogenesis and progression of tumors and many reports have shown that suppression of MYC or MYCN by genetic means results in growth arrest, induction of apoptosis or senescence leading to tumor regression. Knockdown of MYC even results in regression of brain tumors driven by Trp53 and Pten loss in astrocytic cells (67).

In an attempt to attenuate MYCN expression in NB, Galderisi et al. (68) utilized antisense MYCN oligonucleotides, where they demonstrated three-fold decrease in mRNA levels. Subsequently, the reduction on MYCN led to either differentiation or apoptosis, depending on the NB cell type. In another study, von Bueren et al. (69) demonstrated reduced proliferation and clonogenicity, and induced G1 arrest following siRNA-mediated MYC downregulation in DAOY MB cells. Although this may support the idea of tumor cells being addicted to MYC/MYCN signaling, such strategy should be taken with caution, as the authors (69) showed increased resistance to apoptosis and ionizing radiation upon MYC suppression.

Inducible transgenic brain tumor models, where for example *tet*-inducible promoter regulates a transgene, can be utilized to turn on and off cancer genes. We have previously utilized this strategy to first demonstrate the role of MYCN in Group 3 MB development and subsequently showed that long-term withdrawal of MYCN results in tumor regression and life-long remission (70). These tumors likely show robust oncogene addiction as short-term withdrawal of MYCN further showed good efficacy regardless of additional p53 mutations (71).

### Direct MYC/MYCN Inhibitors

A MYC dominant negative gene product, called Omomyc, has a capacity to promote MYC-induced apoptosis (72). In ATRT, Omomyc-mediated MYC suppression led to decreased cell proliferation *in vitro* and *in vivo*, while at the same time significantly prolonging animal survival (73). Similarly, expression of Omomyc in well-established mouse model of glioma (74) prevented tumor formation *in vivo*, reduced proliferative and self-renewal capacity of glioma initiating cells, and lead to mitotic crisis in tumor cells (75). A purified peptide of Omomyc shows further promise *in vivo*. This mini-protein shows sufficient biodistribution to suppress tumor growth in lung cancer models while avoiding toxicity in treated animals (76). As described above, the MYC-MAX protein-protein interaction is required for MYC binding to DNA and poses as a great potential target in MYC and MYCN-driven cancers (77). Recently a MYC-MAX complex inhibitor, MYCMI-6, was described by Castell et al. (78) that not only decreases proliferation and induces apoptosis, but it spares cells with normal levels of MYC. MYCMI-6 is also described to target MYCN-MAX interactions and shows great promise *in vivo* (78). Another example of MYC-MAX inhibition is MYCi975, found in a drug screen with rapid *in vivo* testing for drug efficacy in prostate cancer (44). This drug affects both MYC-MAX protein interaction as well as MYC protein stability and was successfully used in combination with anti-PD1 therapy. MYCi975 is yet to be tested in brain tumor models but decreased MYCN protein levels in a neuroblastoma cell line (44).

The bottleneck for successful treatment of a brain tumor is to be able to deliver a therapy that can circumvent various barriers and efficiently reach the tumor cells in the brain (79, 80). Apart from the normal BBB the brain tumor itself also creates a barrier referred to as the blood-tumor barrier (BTB) that portrays features of non-uniform permeability and active efflux of molecules/drugs that are pumped out (81). It is not clear how well many direct MYC inhibitors or MYC-MAX interaction inhibitors penetrate the BBB/BTB and if they will provide efficacy in malignant brain tumors. Different approaches allowing for more efficient brain tumor delivery of such drugs exist (82). First, direct local delivery of the drug by intrathecal or intraventricular delivery means could be useful were osmotic pumps could provide long-term delivery of drugs. Second, and especially if tumors are considered inoperable (like e.g. DIPGs), convection enhanced delivery can be an option where drugs are directly infused into the parenchyma to promote a forced bulk convective flow into the tumors. Third, focused ultrasound pulses that transiently open up the BBB/BTB could precede delivery of a MYC/MYCN inhibitor. Here, low intensity ultrasound is often combined with circulating microbubbles (made up by lipids, albumin, or polymers) that vibrate in response to the sound to create increased vessel permeability.

### MYCN Transcriptional Machinery

MYC-MAX bind E-BOX sequences in promoters and enhancers as discussed above and recruits the protein complexes needed for transcription and proximal promoter pause release to start elongation (83). In more detail, the acetylated lysine residues on histone tails of open chromatin are bound by bromodomain containing proteins (BRDs) and coactivators of the BET family. The BET family of epigenetic readers consists of BRD2, BRD3, BRD4, and BRDT of which BRD4 is the most studied and understood (84). BRD4 binding recruits the P-TEFb complex made up of CDK9 and binding partner cyclin T that phosphorylates RNA Pol II to engage elongation by pause release (85). Bromodomain and BET inhibitors are so called epigenetic drugs and the BET inhibitors JQ1, described a decade ago by Filippakopoulos et al. (86) and iBET by Nicodeme et al. (87), were proof-of-principle drugs of MYC transcriptional inhibition. JQ1 shows good efficacy in multiple MYC cancers as well as MYCN overexpressed CNS tumors such as MBs (88, 89) and NBs (90). JQ1 and iBET are pan-BRD inhibitors but as BRD4 is often the most dominant BRD in cells these drugs preferentially inhibit BRD4 and sequentially blocks MYC and MYCN dependent transcription. BET inhibitors regularly but not always inhibit the transcription of the MYC/N oncogene itself (91) by competitive binding to the acetyl binding domains of the BET proteins. Though widely used in a laboratory setting, JQ1 was found unfit for clinical applications due to the very short half-life of the drug and numerous efforts have been made to find improved alternative inhibitors of BET that are currently investigated in several clinical trials (92, 93). BET inhibitor resistance is another problem and as for many targeted therapies intracellular re-routing and compensatory mechanisms are likely causes. Finding the mechanisms will help to choose the appropriate drug combination to block any likely escape path for the cancer cells. Targeted nanoparticle delivery of combined JQ1 and temozolomide across the BBB to GBM cells *in vivo* prolonged survival and lowered the systemic drug toxicity in mice (94). However, as the authors discuss, the efficiency of delivery is dependent on the specific surface markers on cancer cells and it requires careful thought and investigation of the individual tumor to design these ligand-targeted nanoparticles (94).

A new generation of BET inhibitors recently emerged, specifically targeting only one of two bromodomains (BDs) on the BRDs (95, 96) in contrast to pan-BRD inhibitors that have equal affinity for both. iBET-BD1, and not iBET-BD2, was found to have similar antiproliferative effects on cancer cell lines as a pan-BRD inhibitor. Also, iBET-BD1 was enough to displace BRDs from chromatin, even at MYC super enhancers in cancer cells (95). By contrast, novel iBET-BD2 compounds still showed good efficacy in MYC-driven pediatric tumors (96). These separate findings need to be further investigated to understand how these inhibitors could be used against MYCN-driven brain tumors.

Targeting the transcriptional machinery is not limited to BET inhibition but there are more traditional strategies using kinase inhibitors that would offer small molecule drugs able to penetrate the BBB. Zotiraciclib is an inhibitor with effects against CDKs, both cell cycle and transcriptional kinases (97) and was recently given orphan drug status in combination with temozolomide for treatment of GBM. Its effect is mainly through inhibition of CDK9, the kinase domain of P-TEFb binding to BRD4 and phosphorylating RNA polymerase II (98) making it principally similar to the successful strategy of targeting MYCN through BET inhibition.

Three-dimensional DNA structures called G-quadruplexes can form in guanine rich regions and do so also in the MYC promoter region. They are two or more secondary structures between tetrads of guanine molecules bound by hydrogen bonds (99). In the c-MYC promoter, stabilization of G-quadruplexes using small molecule inhibitors decreases MYC gene transcription (99–101). Several c-MYC G-quadruplex stabilizers have emerged (102–107), but it is so far unknown if these also target MYCN. However, similar to c-MYC, G-quadruplex structures have been identified near the MYCN promoter region (108). Enniatin B has been found to specifically target MYCN G-quadruplexes (109), which is both promising and discouraging as the more developed drugs targeting c-MYC might be c-MYC specific. This would indicate that MYCN-driven brain cancers have a long way to go in this promising field.

A new strategy deployed for targeting proteins are Proteolysis Targeted Chimeras (PROTACs). PROTACs are bifunctional three-parted drugs with one of the units binding to the protein of interest and one binding to the VHL domain of ubiquitin ligase protein E3. These two units are tethered by a linker to put the E3 ligase in close proximity to the targeted protein for ubiquitylation and subsequent proteasomal degradation (110). The PROTAC compound MZ1 selectively targets BRD4 and fine tuning of this principle lead another group to developed A1874, which is able to degrade BRD4 and at the same time stabilize p53 by binding specifically to the E3 ligase MDM2 (111, 112). To the best of our knowledge, the ability of PROTACs to cross the BBB is not yet known.

### Ribosome Biosynthesis

As MYCN plays an important role in protein synthesis, there has been a growing number of discussions whether the biosynthesis of ribosomes and thus protein synthesis can be exploited in targeting MYC/MYCN dysregulated tumors. Two inhibitors, originally identified as RNA pol I inhibitors, quarfloxin (113) and CX-5461 (114), blocked ribosome synthesis in human MYCN-amplified NB cells, leading to reduction of MYCN protein levels (114). Authors furthermore demonstrated the antitumor effect of CX-5461 *in vivo* opening a new therapeutic avenue for MYCN-amplified tumors. To our knowledge, indirect targeting of MYCN *via* RNA pol I inhibition has not been evaluated, but given its promising efficacy in NB (115), it is worth considering in the future.

### Cell Cycle and MYCN Stability

Tumor cells proliferate rapidly and hijack cell cycle regulation through mutation or deregulation of inherent vital cell cycle promoting and/or safety mechanisms. MYC’s strong correlation to cell proliferation is well known (116–118) and blocking the cell cycle of rapidly dividing cancer cells is a reasonable strategy deployed for treatment of cancer. CDK inhibitors such as Palbociclib, targeting CDK4/6, have shown great success in hormone receptor positive, HER2-negative metastatic breast cancer and have reignited a previous interest in cell cycle inhibition (119, 120). CDK4/6 inhibition can also be used against MYCN-driven tumors to cause a G1 arrest and its effect has been proven in both MB (88) and NB (121, 122). The ability of CDK4/6 inhibitors to penetrate the BBB has been under investigation. Of the three clinically approved CDK4/6 inhibitors palbociclib, ribociclib, and abemaciclib, it is abemaciclib that shows most promise (33). Abemaciclib is now in clinical trials for high-grade and recurrent brain tumors in both children and adults (NCT02644460, NCT03220646).

Three decades ago, CDK2 was in the spotlight for promising drug targets. The interest was dampened when it was shown that CDK2 inhibition was not sufficient to stop proliferation of cancer cells and none of the interphase CDKs are necessary for cell cycle progression as CDK1 was enough to do their job (123, 124). As inhibitor specificity has improved and more is known about CDK2 biology the interest in targeting this kinase has sprouted anew. In addition to CDK2s role in cell cycle commitment it is also one of several kinases that phosphorylates MYC proteins on S62 (125). A few CDK2 inhibitors are currently in clinical trials, however none of these are CDK2 specific. Hence, other CDKs or even classes of proteins could also be involved in their effects. Milciclib is a highly selective CDK2 inhibitor that also has affinity for CDK7/4/5 and tropomyosin receptor kinase A (TrKA) (126). It has been quite successful in clinical trials and is now on Phase II for thymic carcinoma (NCT01011439). The dual role of CDK2 in MYCN-driven brain tumors was shown to successfully target MYCN-driven MB in 2018. Combining milciclib with JQ1 did indeed prolong survival of MYCN-driven MB bearing mice (88). CDK2 was not found to be amplified or overexpressed in the MYCN-driven MB model GTML emphasizing the potential of inhibiting MYCN driven brain tumors with this strategy even at normal levels of CDK2. Also, the aforementioned CDK9 inhibitor zotiraciclib has affinity and inhibitory effects on CDK2. Both milciclib and zotiraciclib penetrates the BBB making them highly interesting to study further in MYCN-driven brain tumors, perhaps even in combination.

### Aurora Kinases

A family of serine/threonine kinases, named Aurora, plays an important role in regulation of key steps in cell division. They are involved in organization of centrosomes, condensation of chromatin, chromosome attachment to microtubules, and establishment of metaphase plate (127). Aurora kinase A (encoded by AURKA) is aberrantly expressed in many cancers (128), including GBM (129–132), making it a plausible candidate for a targeted GBM therapy. Expression of both AURKA and AURKB (Aurora kinase B) is tightly regulated by MYC transcription factor (133). Moreover, Aurora kinases A and B directly phosphorylate MYC to promote its stabilization and increase its transcriptional activity (134, 135). In pediatric NB and MB, transcription factor MYCN binds Aurora kinase A, thus attenuating G2/M arrest and stabilizing MYCN protein (136), and conversely inhibition of Aurora kinase A promotes MYCN degradation and cell death (71, 137, 138). These findings highlight the importance of Aurora kinases as druggable targets, particularly in tumors which are driven by aberrant MYC/MYCN signaling. In this section of the review, we will further explore therapeutic potential of Aurora kinase inhibitors in brain tumor therapy.

Alisertib is a second generation, ATP competitive Aurora kinase A inhibitor, which inhibits autophosphorylation at T288. Combined Alisertib and BRD4 inhibition results in synergistic decrease of viability in high-risk, MYCN amplified NB cells (139). Alisertib shows also an advantage in pediatric GBM, where *in vitro* effects were observed in a number of patient-derived cells and *in vivo*, by prolonging mouse survival (140). However, emergence of AURKA negative and CD133 positive cells results in relapse *in vivo*, which suggests a need of dual inhibition to overcome resistance. We have previously showed that AURKA inhibition together with BRD4 inhibition successfully inhibits a number of patient-derived GBM cells (141). Interestingly, GBM cells that were most sensitive to AURKA inhibition were those with high level of MYCN expression, although we must emphasize that the combined AURKA and BRD4 inhibition shows strong synergistic antitumor activity in all evaluated GBM cells, irrespective of MYCN levels (141).

Alisertib inhibition in a MYCN-driven model of group 3 MB (70) disrupts AURKA-MYCN complex and inhibits cell viability both *in vitro* and *in vivo* (71, 142). The inhibition of tumor growth was exercised through nearly completed reduction of MYCN protein expression, cell cycle arrest in G2/M phase, but not apoptosis, which is indicative of AURKA inhibition.

Aurora kinase A, among other functions, regulates MYC/MYCN protein stability. Unlike many inhibitors that target Aurora A activity, Gustafson et al. (138) developed a conformation-specific compound CD532 that binds to Aurora A, destabilizes MYC/MYCN and targets them for proteasomal degradation. Although developed in MYCN-amplified neuroblastoma models, this compound shows promising effects on cell cycle and MYC/MYCN stability.

### Upstream Regulation of MYCN *via* PI3K and mTOR

Phosphoinositide 3-kinases (PI3K) regulates MYCN stability through AKT and GSK3β in cerebellar neuron precursors (143, 144), which suggests that MYCN effects can be counteracted by inhibiting upstream MYCN signaling. Indeed, authors (143) demonstrated a substantial loss of wild-type MYCN upon PI3K inhibitor wortmannin, while mutant MYCN^T50A^ and MYCN^S54A^ levels remained unchanged. Similarly, in MYCN-driven models of NB, Cage et al. (145) showed ablation of MYCN following the treatments with two different PI3K inhibitors PIK-75 and PW-12. Furthermore, in mouse allografts of SHH MB authors (145) demonstrated uniform absence of MYCN, reduced proliferation and vascularity, as well as increase of apoptosis following *in vivo* treatment with PW-12, altogether resulting in a significant, more than five-fold decrease in tumor volume.

MYCN is indirectly regulated by upstream signaling mediated through e.g. mammalian target of rapamycin complexes (mTORC), which regulates cell growth and protein synthesis (as reviewed in (146). Over the past decade several mTOR inhibitors have been developed and proven successful in many different cancer models [as reviewed in (147)]. First generation mTOR inhibitors rapamycin and its ester analogue CCI-779 (Temsirolimus) not only inhibited growth and induced cell death in several MYCN-amplified NB cells, but also significantly reduced MYCN protein levels (148). Similarly, another group identified dual PI3K/mTOR inhibitor, NVP-BEZ235 (Dactolisib), to specifically destabilize MYCN proteins in MYCN-dependent tumors (149). More recently, our group has proved that many of such second generation mTOR inhibitors are indeed successful in inhibiting MYCN-amplified SHH MB tumor models both *in vitro* and *in vivo* (52). RapaLink-1, a bivalent third generation mTOR inhibitor, which combines rapamycin with INK128 (Sapanisertib) by an inert chemical linker, has also shown great efficacy in MYCN-driven brain tumor models (150). Several mTOR inhibitors have already been approved or are currently undergoing clinical trials (151), making mTOR inhibition a very promising therapeutic avenue for MYCN-deregulated brain tumors. Especially for SHH-dependent medulloblastoma where more direct SHH pathway drugs, including SMO inhibitors, are shown to induce severe side effects in young children or infants (152). For instance, mTOR inhibitors, such as everolimus, are well tolerated in children treated for epilepsy (153), which is in line with findings of Wu et al., where young mice treated with the BBB penetrable mTOR inhibitor, Sapanisertib showed no toxicity against cerebellar development (154). In **Table 1**, we present a list of selected potential drugs for MYC/MYCN targeted therapy discussed in this paper where the progress of these in preclinical and clinical research is summarized.

**Table 1:** Drugs and compounds for targeting of MYCN signaling.

Inhibitor	Tumor type	Target	Phase	Reference
*Direct MYC/MYCN inhibitors*				
Omomyc	Glioma, ATRT	MYC proteins	Preclinical	(1–5)
MYCMI-6	Various cancers, NB	MYC proteins	Preclinical	(6)
*Inhibitors of MYCN transcriptional machinery*				
JQ1 I-BET	Various cancers, MB, and NB	BRD4	Preclinical	(7–10) (11)
Zotiraciclib	GBM	CDK9	Clinical orphan drug	(12)
Enniatin B	N/A	MYCN	Biochemical	(13)
MZ1	N/A	BRD4	Biochemical	(14)
A1874	Colon cancer, lung cancer, osteosarcoma	BRD4	Preclinical	(15)
*Cell cycle related inhibitors targeting MYCN*				
Palbociclib	MB, NB	CDK4/6	Preclinical	(9, 16, 17)
Abemaciclib	DIPG, brain tumor (NOS), NB, ATRT	CDK4/6	Clinical trial	(19), NCT02644460, NCT03220646
Milciclib	MB	CDK2	Preclinical	(9)
	Thymic carcinoma		Clinical trial	NCT01011439
Alisertib	GBM, MB, NB	Aurora A	Preclinical	(20–23)
	High-risk AML		Clinical trial	NCT02560025
*PI3K/AKT/mTOR inhibitors targeting MYCN*				
PIK-75, PW-12	MB	PI3K	Preclinical	(24)
Rapamycin	NB	mTORC1	Preclinical	(25)
Temsirolimus	NB	mTORC1	Preclinical	(25)
	CNS tumors		Clinical trial	NCT00003712
NVP-BEZ235	MYCN-dependent tumors	mTORC1/2, PI3K	Preclinical	(26)
	Breast cancer		Clinical trial	NCT00620594
Everolimus	Breast cancer	mTORC1	Clinical trial	NCT01783444
	Pediatric epilepsy		Approved	(27)
Sapanisertib	MB	mTORC1/2, PI3K	Preclinical	(28)
*Ribosome biosynthesis inhibitors targeting MYCN*				
Quarfloxin, CX-5461	MYCN-driven NB	RNA Polymerase I	Preclinical	(29)

A list of a selection of drugs or compounds identified as potential direct or indirect targets of MYC/MYCN-driven CNS/PNS tumors. Compounds in the clinical development for another tumor type (and not for brain tumors/CNS tumors) are mentioned in cases where they showed promising results in preclinical CNS tumor models.

### The OCT4/mTOR Malignancy Axis

**Figure 3:**
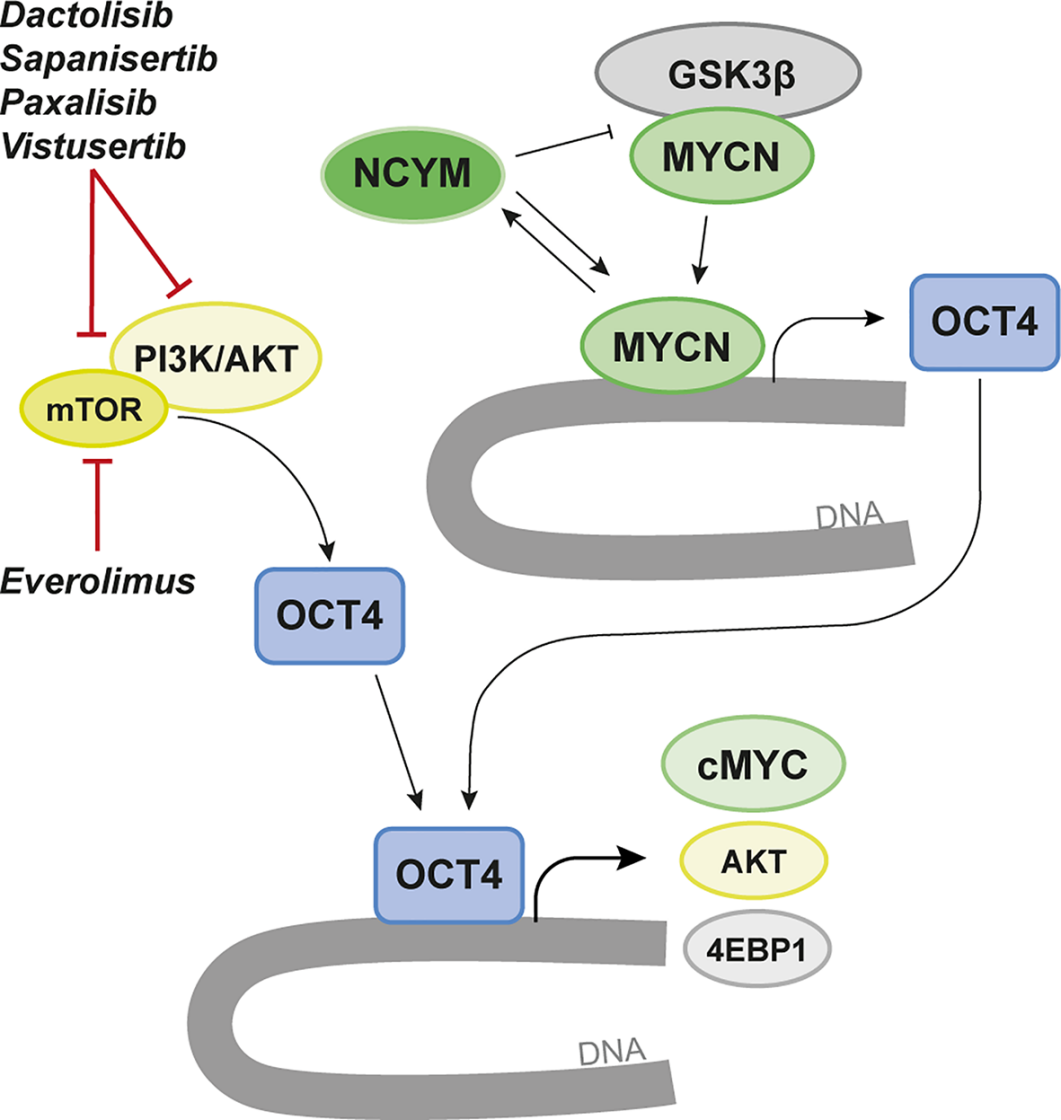
Targeting of MYCN-dependency *via* the OCT4/mTOR Axis. MYCN transcriptionally regulates OCT4 and promotes increased OCT4 levels that correlate with poor prognosis in various brain tumor entities. OCT4 has been found to form a positive regulatory loop that induces increased protein stability of MYCN by increasing the levels of its cis-antisense gene NCYM. In parallel, mTOR/PI3K/AKT promotes OCT4 levels in both normal and malignant cells. Here AKT is known to activate OCT4 by phosphorylation leading to OCT4-dependent upregulation of 4EBP1 and cMYC but also to a positive loop that again promotes AKT expression. We propose a strategy where targeting of OCT4 using various mTOR/PI3K inhibitors could regulate MYCN and suppress MYCN-dependent brain cancer. A few selected inhibitors tested in the brain tumor papers discussed in the review are highlighted.

mTOR is known to promote octamer-binding transcription factor 4 (OCT4) levels in embryonic stem cells (155). In MYCN-driven human brain tumor models generated from primary embryonic or induced pluripotent stem cell (iPSC)-derived neural stem cells we could show a significant correlation of mTOR pathway activation and OCT4 levels (52). When we overexpressed OCT4 we further found increased mRNA levels of 4EBP1 gene (EIF4EBP1) as well as elevated phosphorylation of 4EBP1 that marks mTOR pathway activity downstream of mTORC1. The OCT4/mTOR axis correlated with poor prognosis in SHH MB patients and OCT4-overexpression increased the malignancy of these pediatric brain tumors (**Figure 3**).

OCT4 has previously been shown to increase metastasis and malignancy in MB cell lines (156) and malignancy in GBM where AKT is activating OCT4 (157). OCT4 phosphorylation at T235 by AKT is increasing OCT4 stability and correlate with apoptotic resistance and tumor malignancy (158).

OCT4 has an important regulatory role in MYCN-amplified tumors (**Figure 3**). In MYCN-driven NB OCT4 was found to induce increased levels of MYCN by increasing the levels of its cis-antisense gene NCYM (159). Subsequently, NCYM is stabilizing MYCN by inhibiting GSK3β to protect MYCN from proteasomal degradation (160). In this auto-regulatory loop MYCN can again induce OCT4 and other stem-cell related genes. NCYM correlates with OCT4 levels and with poor prognosis in MYCN-amplified tumors. Various inhibitors of mTOR and/or PI3K/AKT can suppress the OCT4/mTOR axis in malignant brain tumors (52).

At another dimension which might be of importance for treatment resistance in MYCN-driven cancer, involves OCT4 phosphorylation at S111 *via* MAPKAP2 that can promote MYC expression (**Figure 3**). This might help identifying a therapy-resistance mechanism in MYCN-driven NB, providing an escape route driven by OCT4-activated MYC (161) in recurrent tumors.

## Summary and Discussion

MYC family members are found overexpressed in more than half of all cancers highlighting its role as one of the most important oncogenes. MYC proteins are involved in brain tumor initiation, maintenance and progression in both children and adults. MYCN has an important role also in normal brain development. It is known that misregulation of its expression occurs during early development in childhood neoplasms and that MYCN is likely activated during progression in adult brain tumors. While several ways of targeting MYCN is approaching and show promise, there are still many obstacles regarding delivery of direct and indirect ways to target this transcription factor. Better ways and tools to deliver MYCN-targeting drugs that penetrates the BBB is needed using e.g. chemical modifications of substances, nano-particles as drug carriers, or ultrasound technology for temporal opening of the BBB to mediate efficiently high concentrations of the MYCN drug at the tumor site.

While many drugs target cMYC it is important to investigate if they are also relevant for MYCN-driven tumors or if they can be modified to target MYCN specifically to avoid unnecessary side effects. Therefore, it is of utter importance that published data on cMYC targeting also get tested in relevant MYCN-driven cancer cell and mouse models. Appropriate animal brain tumor models should not only be used to test and determine efficacy of novel drugs but will also be valuable in observing tolerability and evident toxicities of the tested compound in the preclinical evaluation. These animal models are useful tools for early detection of side effects from drugs on the normal growth of animals and on their proper brain development or consideration of future use in infant and pediatric brain tumor patients.

As many before have suggested, combination treatments are most likely the best way to circumvent acquired drug resistance. Understanding the mechanisms behind both the drug effect and future resistance will help deciding efficient drug combinations. On this topic, one should also consider that we could shorten the bench to bedside time frame by including relevant standard treatments in preclinical testing of potential MYCN drugs. *In vivo* testing of MYCN drugs for brain tumors should include irradiation and chemotherapy similar to what is used in the clinic to get solid data with a better chance to succeed in affected patients. By simply getting a drug into clinical trials it could benefit specific patients. We stay optimistic and believe that any of these measures will help providing better responses and hopefully even a cure for these devastating malignancies.

## Author Contributions

AB and FS wrote the majority of the review with extensive contributions from MC and SH. AB, SH, and FS made the figures with input from MC. MC made the table. All authors contributed to the article and approved the submitted version.

## Conflict of Interest

The authors declare that the research was conducted in the absence of any commercial or financial relationships that could be construed as a potential conflict of interest.
